# Risk prediction models for postoperative intra-abdominal infection in patients with digestive system tumors: a systematic review

**DOI:** 10.3389/fonc.2025.1678860

**Published:** 2025-11-18

**Authors:** Yu Wang, Li Wang, Yunhong Du, Jianwei Li, Yao Shi, Zhiyuan Zhang, Lili Peng

**Affiliations:** 1 School of Nursing, Hunan University of Chinese Medicine, Changsha, China; 2 Department of Nursing, Qingdao Hiser Hospital, Qingdao, China; 3 Cardiovascular Surgery Intensive Care Unit, The First Affiliated Hospital of Naval Medical University, Shanghai, China

**Keywords:** postoperative intra-abdominal infection, digestive system, malignant tumors, risk prediction model, systematic review

## Abstract

**Background:**

Postoperative intra-abdominal infection (IAI) is a severe complication in digestive system tumor patients, increasing hospital stays, costs, and mortality. Accurate prediction enables early intervention and better prognosis. However, existing prediction models lack comprehensive evaluation due to diverse study designs, data sources, and assessment methods. A systematic review is needed to develop a structured prediction model for postoperative IAI in patients with digestive system tumors, and to provide references for the optimization or development of such prediction models in the future.

**Methods:**

A computerized search was conducted for relevant studies in PubMed, Web of Science, Embase, Cochrane Library, CINAHL, CNKI, CBM, WanFang Data and VIP databases, with the search time restricted to the establishment of the database to 6 February 2025. Literature screening was performed independently by two researchers and data information was extracted, and the risk of bias and applicability of the model were evaluated using PROBAST.

**Results:**

A total of 22 studies with 9,127 patients were included in the literature. The area under the operating characteristic curve (AUC) of the subjects included in the model ranged from 0.702 to 0.987, and the predictive performance of the model was good in all cases (AUC >0.700). Three cases were internally validated, two cases were externally validated, and two cases were evaluated using a combination of internal and external validation for the model. The most common predictors included length of surgery, comorbid diabetes mellitus, serum albumin level, length of drain retention, and age.

**Conclusions:**

Currently, the prediction model for the risk of postoperative IAI in patients with digestive system tumors is still in the research and development stage. Based on the PROBAST assessment, all studies were considered to have a high risk of bias. Subsequent studies should refer to the reporting guidelines of the PROBAST. Additionally, they should focus on large sample sizes and rigorously designed multicenter external validation to further evaluate the efficacy and feasibility of the models in clinical practice.

## 1 Introduction

GLOBOCAN statistics showed that (1) there were about 4.98 million new cases and 3.25 million deaths of digestive system tumors worldwide in 2022. According to the malignant tumor registry data (2, 3), the annual number of new cases and annual number of new deaths of digestive system tumors in China are ranked No. 1 among all cancer types, which has become a major public problem that seriously threatens the health of people.

At present, surgery, as one of the core treatment modalities for digestive system tumors, implements precise resection of the lesion tissue, which can effectively block the invasive and metastatic pathways of tumor cells and prolong the survival time of patients. A serious complication after surgical treatment of digestive system tumors is intra-abdominal infection (IAI) (4), which is a key factor in the occurrence of secondary surgery and postoperative death (5) and its occurrence can lead to a significant prolongation of patients' hospital stay, which can have a serious negative impact on their postoperative recovery and quality of life (6). It can even increase the proliferation rate and migration ability of tumor cells in patients (7), threatening the life safety of patients. How to reduce the high incidence of postoperative IAI in patients with digestive system tumors is an urgent clinical problem.

Risk prediction model is based on multiple predictive variables to establish a statistical model and predict the probability of the occurrence of the relevant outcome events (8, 9), which can help healthcare professionals to accurately identify high-risk groups at an early stage and carry out predictive interventions, thus reducing the incidence of specific adverse events while improving the prognosis of patients and realizing the rational use of medical resources. Nowadays, a variety of prediction models for postoperative IAI in patients with digestive system tumors have been developed both at China and abroad, but their quality and predictive performance are not the same, and their clinical applicability still needs to be improved.

In this study, we searched for domestic and international studies on postoperative IAI risk prediction models for patients with digestive system tumors. It focused on diagnostic performance, sensitivity, and specificity, and conducted a comprehensive analysis of the risk bias and clinical applicability of the models, aiming to provide a scientific basis for the development of modeling and clinical application of such models in the future.

## 2 Methods

### 2.1 Study design

This study was conducted following the guidelines from PRISMA, ensuring transparent and comprehensive reporting of methods and results. Additionally, the study has been registered with PROSPERO (ID : CRD42024564772). As this study entails meta-analysis and systematic review of previously published research, ethical approval was deemed unnecessary.

### 2.2 Search strategy

A standardized search of PubMed, Web of Science, Embase, Cochrane Library, CINAHL, CNKI, CBM, WanFang Data, and VIP databases was performed to identify studies on postoperative IAI risk prediction models for patients with gastrointestinal tumors, with the search time restricted to the establishment of the database to 6 February 2025. To ensure methodological rigour, non-peer-reviewed materials (e.g., editorials, letters, preprints) were excluded during screening. Institutional affiliations and DOI numbers were verified to confirm formal publication status. The search strategy included three primary terms: “Digestive System Neoplasm”, “Intra-Abdominal Infection”, and “prediction Model”. Retrieval is conducted using a combination of subject terms and free terms, as well as the literature tracing method. Additionally, reference lists of selected articles were manually reviewed to capture further relevant studies.

### 2.3 Inclusion and exclusion criteria

Inclusion criteria.

- The cases were patients with digestive system tumors aged 18 years and above;
- The study focused on the construction and/or validation of postoperative IAI prediction model for patients with digestive system tumors;
- The types of studies included prospective, retrospective, and cross-sectional studies, etc;
- Literature in Chinese and English.

Exclusion criteria.

- The full text was not available;
- Only analyzing the risk factors of postoperative IAI in patients with digestive system tumors without constructing a model;
- Model predictors ≤2;
- The information was incomplete, the indexes that could not be extracted, or the publication was duplicated.

### 2.4 Literature screening and data extraction

The research team consists of Masters of Nursing (3 members), experts in evidence-based nursing (2 members), and experts in emergency nursing (2 members); all members have completed the study of evidence-based nursing courses. The screening process of this study comprises two stages: title/abstract screening and full-text screening, both of which are jointly conducted by all members of the research team. At the abstract, title or full paper stage, a detailed check of each study is carried out by pairs of authors. Any disagreements are resolved through reaching a consensus and/or, if necessary, involving last author for arbitration to determine the final evaluation conclusions.

The screening process begins with removing duplicate records, followed by conducting preliminary screening of the titles and abstracts of all records to exclude entries that do not meet the inclusion criteria. Each record is classified into three categories: 'include', 'exclude', or 'maybe', and each classification result is accompanied by notes, which serve to identify relevant literature and exclude irrelevant literature. For questionable literature, the research team does not directly exclude it at this stage but retains it for the next screening stage for full-text review. For all records that have passed only the title and abstract screening and meet the inclusion criteria, their full texts are obtained before conducting the final screening; for studies that do not meet the inclusion criteria, the reasons for their exclusion are documented in detail.

According to the checklist for critical appraisal and data extraction for systematic reviews of prediction modelling studies (CHARMS) proposed by Moons et al. (10), a data extraction table was developed. Two investigators independently extracted data including the first author, publication year, country, study design, study subjects, data source, diagnostic method for predicted outcomes, modelling methods, variable selection, sample size, handling of missing data, method for handling continuous variables, final predictors, model performance, validation method, model presentation. It was independently conducted by 2 researchers who had all received evidence-based training. After extracting the data, cross-checking was performed; in case of disputes, consultation was carried out with a third researcher.

### 2.5 Evaluation of risk of bias and applicability of the included studies

The risk of bias and applicability of the included studies were comprehensively evaluated using the prediction model risk of bias assessment tool (PROBAST) (11). Prior to the initiation of literature evaluation, all evaluators in the research team were provided with systematic review training. Upon the completion of training, a simulation evaluation test was used to assess the evaluators' mastery of the training content. Only those evaluators who passed the test were eligible to participate in the formal evaluation work, so as to ensure the reliability and consistency of the evaluation results. The literature evaluation was independently conducted by the first and second authors who specialize in oncology nursing and have received evidence-based training; cross-verification of their respective evaluation results was also carried out. When discrepancies or doubts emerged in the evaluation opinions, the last author was involved for joint discussion. If necessary, the root causes of the discrepancies were traced and analyzed, and a consensus conclusion was reached through group discussion.

In addition, we used the TRIPOD reporting guidelines to assess the reporting quality of each included study. The adherence of each study to the 22 items was independently evaluated by first and second authors, and the TRIPOD compliance of each study was expressed as the number and percentage of reported items.

### 2.6 Statistical analysis

In this study, the area under the receiver operating characteristic curve (AUC) was chosen as an indicator of discrimination ability. AUC was pooled by a random-effects model to assess overall discrimination across all prediction models and across clinical settings. Meta-analysis was performed using Stata 16.0 software. The odds ratio (OR) and 95% confidence interval (95% CI) were used as effect size statistics. The *Q* test and *I*² heterogeneity index were applied to assess the presence of heterogeneity: If *I*² <50% and *P >*0.1, the homogeneity was considered acceptable, and a fixed-effects model was used for analysis; if *I*² >50% and *P <*0.01, further sensitivity analysis was conducted; if heterogeneity could not be eliminated, a random-effects model was adopted for analysis. A *P <*0.05 was considered statistically significant.

## 3 Results

### 3.1 Literature screening process and results

**Figure 1:**
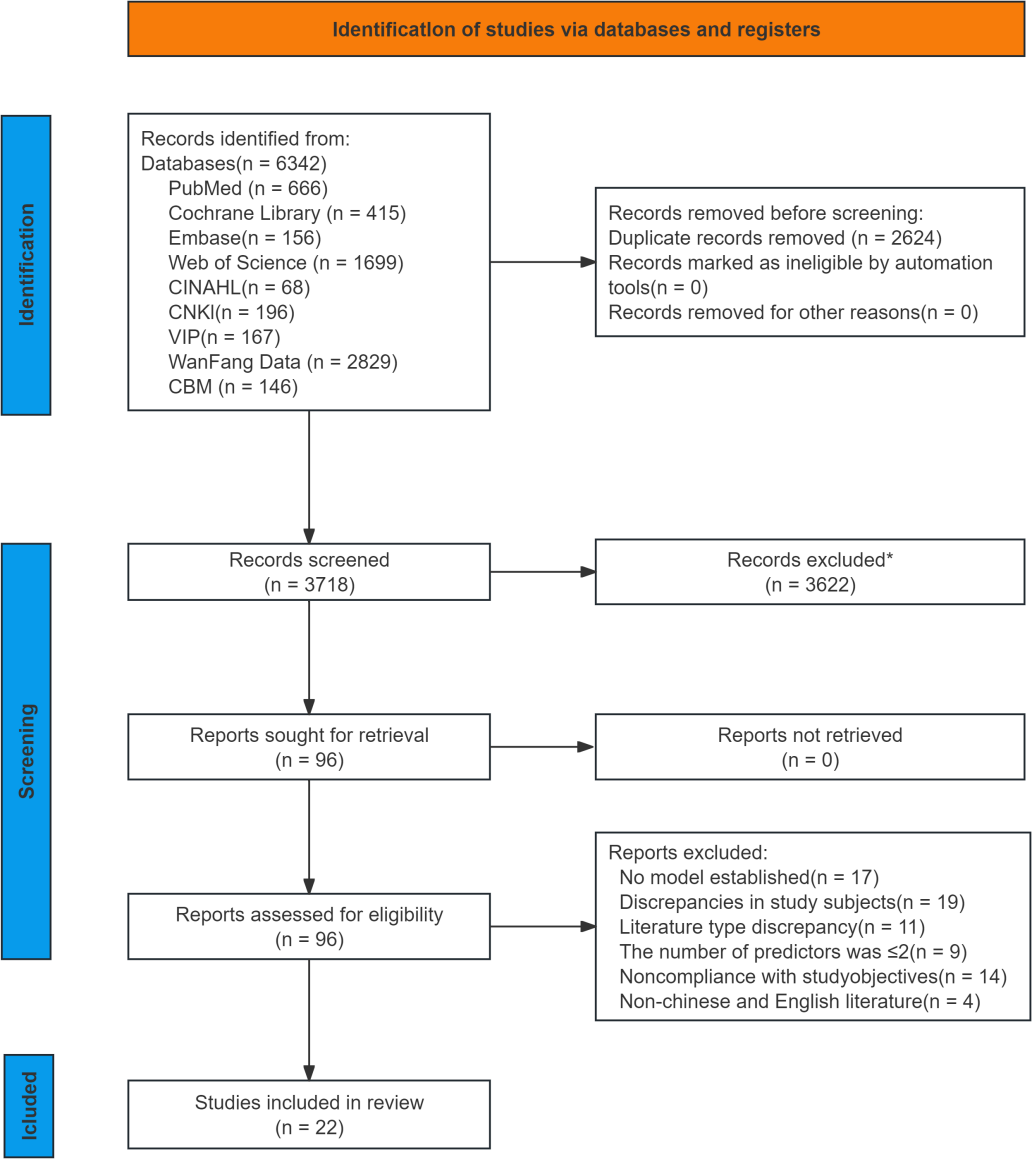
Flow chart of literature screening.

In this review, 6,342 relevant studies were retrieved through preliminary screening. After duplicate records were removed using EndNote software, 3,718 records remained. A further 3,622 studies were excluded after reading the titles and abstracts. Following this process, 96 studies were selected for full-text retrieval, and those that met the following exclusion criteria were excluded: no model was established, mismatched research purposes, inappropriate study subjects, insufficient predictors, and inconsistent literature types. Finally, 22 studies (12–33) were included in this review. Overall, the PRISMA flow diagram (**Figure 1**) of this study demonstrates the rigorous process of selecting studies that meet the inclusion criteria for the systematic review.

### 3.2 Basic characteristics of the included studies

The total number of samples included in the literature was 9,127 cases, and the number of outcome events ranged from 15 to 194 cases; the studies were published between 2004 and 2023, of which 19 studies (15–33) were in the last 5 years; three studies (12, 14, 17) were prospective study designs, and the remaining were retrospective studies. The basic characteristics of the included studies were shown in **Table 1**.

**Table 1:** Basic characteristics of the included studies (n = 22).

Studies	Country	Study design	Participants	Data source	Diagnostic approach for predicting results
Mao CC (12)	China	Prospective	Patients undergone radical gastrectomy for gastric cancer	The First Affiliated Hospital of Wenzhou Medical University	Diagnostic criteria for nosocomial infection (Trial)
Chen L (13)	China	Retrospective	Patients undergoing radical gastric cancer surgery	Jiangsu Provincial People's Hospital	Diagnostic criteria for intra-abdominal infection after gastrointestinal surgery (2015 edition)
Ye GQ (14)	China	Prospective	Patients undergoing radical colorectal cancer resection	The Second Affiliated Hospital of Wenzhou Medical University and Pingyang County Hospital of Traditional Chinese Medicine	Clinical features were combined with imaging or endoscopic findings
Hu LS (15)	China	Retrospective	Patients undergoing pre-cancer rectal resection	The Second Affiliated Hospital of Guilin Medical College	Clinical features were combined with imaging or endoscopic findings
Cagigas (16)	Spain	Retrospective	Patients undergoing surgery for colorectal cancer	Hospital Universitario Marqués de Valdecilla	Clinical features were combined with imaging or endoscopic findings
Sun C (17)	China	Prospective	Patients undergoing elective radical surgery for advanced digestive system cancer	Peking Union Medical College Hospital	Diagnostic criteria from the Centers for Disease Control and Prevention (CDC)
Liu SF (18)	China	Retrospective	Patients with anastomotic fistula after radical surgery for colorectal cancer	Luoyang Central Hospital affiliated to Zhengzhou University	Chinese guidelines for the diagnosis and treatment of intra-abdominal infections (2019 edition)
Pei G (19)	China	Retrospective	Patients undergoing radical surgery for colorectal cancer	Zhongda Hospital, Southeast University	Clinical features were combined with imaging or endoscopic findings
Zhang Y (20)	China	Retrospective	Gastric cancer resection patients	The First Affiliated Hospital of Wenzhou Medical University	Clinical Diagnosis of Intra-abdominal Infection
Liu J (21)	China	Retrospective	Patients undergoing surgery for colorectal cancer	The First Hospital of Qinhuangdao	Unspecified
Luo J (22)	China	Retrospective	Patients undergoing radical gastric cancer surgery	Affiliated Hospital of Qinghai University	Chinese Guidelines for the diagnosis and treatment of intra-abdominal infection (2019 edition) and Diagnostic Criteria for Nosocomial Infection (Trial)
Wu TL (23)	China	Retrospective	Patients undergoing radical gastric cancer surgery	Jiangxi Provincial People's Hospital	Chinese guidelines for the diagnosis and treatment of intra-abdominal infections (2019 edition)
Yang L (24)	China	Retrospective	Patients undergoing resection for hepatocellular carcinoma	The Second Affiliated Hospital of Shandong First Medical University	Diagnostic Criteria for Nosocomial Infection (Trial)
Zhang WB (25)	China	Retrospective	Patients undergoing surgery for colorectal cancer	Baotou Cancer Hospital	Guidelines for Prevention and Control of Nosocomial Infections
Chen YG (26)	China	Retrospective	Patients undergoing surgery for colorectal cancer	Jiamusi tumor (tuberculosis) hospital	Diagnostic Criteria for Nosocomial Infection (Trial)
Jin J (27)	China	Retrospective	Patients undergoing radical surgery for colorectal cancer	Affiliated Hospital of Southwest Medical University	Chinese guidelines for the diagnosis and treatment of intra-abdominal infections (2019 edition)
Luo C (28)	China	Retrospective	Patients undergoing resection for hepatocellular carcinoma	Hangzhou Xixi Hospital	Chinese guidelines for the diagnosis and treatment of intra-abdominal infections (2019 edition)
Shi ZQ (29)	China	Retrospective	Patients undergoing surgery for colorectal cancer	Dongying District People's Hospital of Dongying city	Diagnostic Criteria for Nosocomial Infection (Trial)
Sun Y (30)	China	Retrospective	Gastric cancer resection patients	Qingdao Municipal Hospital	Diagnostic Criteria and Surveillance Techniques of Nosocomial Infection
Wang S (31)	China	Retrospective	Patients undergoing resection for gastrointestinal malignant tumors	Suqian First People's Hospital	Clinical features were combined with imaging or endoscopic findings
Liu CQ (32)	China	Retrospective	Patients undergoing radical surgery for colorectal cancer	Affiliated Suzhou Hospital of Anhui Medical University	Unspecified
Yu X (33)	China	Retrospective	Elderly patients undergoing radical gastrectomy for gastric cancer	Dandong Central Hospital of China Medical University	Chinese Medical Association guidelines for the clinical diagnosis and treatment of gastric cancer

### 3.3 Establishment of risk prediction models

A total of 23 models were constructed. One study (16) used multiple interpolation to handle missing data, seven studies (12, 14, 17, 19, 20, 27, 28) did not report the method for missing data, and the remaining studies excluded subjects with incomplete data when determining the inclusion and exclusion criteria; in terms of modeling methods, one study (22) chose Bayesian network for modeling, one study (16) carried out both logistic regression and Bayesian network modeling, and the remaining studies adopted logistic modeling; as for model presentation, eleven models (12, 15, 19, 20, 23, 27–29, 31–33) were finally presented as nomogram, two studies (21, 24) did not report the form of model presentation, and two studies (16, 22) presented models through Bayesian network, and the rest were presented as risk scoring formulas or scoring systems.

Each model finally incorporated 3–6 predictors, which were further analyzed and summarized into the following 4 categories: patient body-related factors include age, comorbid diabetes mellitus, tumor size and so on; surgery-related factors include length of surgery, combined organ resection, length of retention of drains and so on; nutritional status-related factors include serum albumin (ALB), sarcopenia, subcutaneous fat content and so on; laboratory-related indicators include calcitoninogen, systemic immunoinflammatory index, body mass index and so on. The most frequent predictors were length of surgery (n=10), ALB (n=8), comorbid diabetes mellitus (n=8), length of drain retention (n=4), age (n=4), and so on. The basics of the modeling are shown in **Table 2**.

**Table 2:** Basic information of the risk prediction model.

Studies	Modeling method	Selection of variables	Sample size		Methods for handling missing values	Continuous variable treatment methods	Included predictors
			Negative events	Positive events			
Mao CC (12)	LR	Single + Multiple	621	61	Not mentioned	Categorical variables	Tumor diameter, combined organ resection, pathological type, and sarcopenia
Chen L (13)	LR	Single	620	63	Exclusion	Continuous variables	Body temperature, heart rate, white blood cell count, abdominal pain, abdominal distention
Ye GQ (14)	LR	Single + Multiple	339	44	Not mentioned	Categorical variables	Sarcopenia, tumor diameter, and age
Hu LS (15)	LR	Single + Multiple	215	32	Exclusion	Categorical variables	Diabetes mellitus, duration of surgery, anastomotic leakage, and pulmonary infection
Cagigas (16)	LR, BN	Unspecified	450	194	Multiple imputation	Continuous variables	Type of surgical anastomosis, surgical method, PCT, CRP
Sun C (17)	LR	Single + Multiple	788	51	Not mentioned	Continuous variables	Gastrectomy, colorectal resection, pancreaticoduodenectomy, duration of anesthesia, and duration of ICU stay
Liu SF (18)	LR	Single + Multiple	51	21	Exclusion	Categorical variables	Diabetes mellitus, duration of surgery, ALB, and duration of antibiotic use
Pei G (19)	LR	Single + Multiple	356	46	Not mentioned	Categorical variables	ALB, LWR, subcutaneous fat content, skeletal muscle mass
Zhang Y (20)	LR	Single + Multiple	438	34	Not mentioned	Continuous variables	Hypertension, combined organ resection, history of abdominal surgery, and duration of surgery
Liu J (21)	LR	Single	44	52	Exclusion	Continuous variables	SII, neutrophil CD64, CD11b
Luo J (22)	BN	Single + Multiple	1279	169	Exclusion	Categorical variables	Age, duration of surgery, number of drainage tubes, vascular invasion, and smoking history
Wu TL (23)	LR	Single + Multiple	301	31	Exclusion	Categorical variables	ASA grade, BMI, comorbidity, ALB, pTNM stage, NEUT
Yang L (24)	LR	Single + Multiple	86	20	Exclusion	Categorical variables	Age, diabetes, ALB, Hb, duration of operation and duration of drainage tube indwelling
Zhang WB (25)	LR	Single + Multiple	388	55	Exclusion	Continuous variables	Diabetes mellitus, duration of surgery, Y*ap* mRNA, *ta*Z mRNA, *mst*1 mRNA levels
Chen YG (26)	LR	Single + Multiple	151	45	Exclusion	Categorical variables	Diabetes mellitus, preoperative intestinal obstruction, length of incision, duration of operation, duration of drainage tube indwelling, and length of hospital stay
Jin J (27)	LR	Single + Multiple	299	55	Not mentioned	Categorical variables	Preoperative complications included intestinal obstruction, hyperglycemia and metabolic syndrome
Luo C (28)	LR	Single + Multiple	410	56	Not mentioned	Categorical variables	Age, diabetes mellitus, duration of surgery, duration of drainage tube indwelling, and ALB
Shi ZQ (29)	LR	Single + Multiple	136	43	Exclusion	Categorical variables	Diabetes mellitus, ALB, postoperative stoma, and duration of drainage tube indwelling
Sun Y (30)	LR	Single + Multiple	438	43	Exclusion	Categorical variables	Tumor diameter, duration of surgery, ALB, lymphovascular invasion
Wang S (31)	LR	Single + Multiple	177	23	Exclusion	Categorical variables	Abdominal pain, abdominal distention, PCT, body temperature, and white blood cell count
Liu CQ (32)	LR	Single + Multiple	65	15	Exclusion	Continuous variables	NLR, PLR, SII, CEA
Yu X (33)	LR	Single + Multiple	295	27	Exclusion	Continuous variables	BMI, Glu, Hb, ALB, operation time, blood loss

LR, logistic regression; BN, Bayesian network; Single, single factor analysis; Multiple, multiple factor analysis; ASA classification, American Society of Anesthesiologists health status classification; CRP, C-reactive protein; PCT, procalcitonin; Y*ap* mRNA, Yes-associated protein mrna; *ta*Z mRNA, transcriptional coactivator PDZ-binding motif; *mst1* mRNA, mammalian STE20-like protein kinase 1; LWR, lymphocyte-leukocyte ratio; PNI, prognostic nutritional index; CEA, carcinoembryonic antigen; NEUT, percentage of neutrophils; CA242, carbohydrate antigen 242; NLR, neutrophil-lymphocyte ratio; PLR, platelet-lymphocyte ratio; SII: systemic immune inflammation index; BMI, body mass index; Glu, glucose; Hb, hemoglobin; ALB, Serum albumin.

### 3.4 Model predictive efficacy

The discriminative ability of the models involved in this study was mainly evaluated by AUC or the concordance index (C-index) of the subjects' work characteristics, and four studies (14, 15, 23, 27) evaluated the model discriminative ability by using both the AUC and C-index, one study (12) used the C-index, and the rest of the studies reported the discriminative ability using the AUC; except for Luo J et al. (22) who did not report the discriminative ability, the rest of the studies were >0.7, which represents a high predictive performance; 15 studies (13, 15, 17–19, 23, 25–33) reported calibration methods, including Hosmer-Lemeshow goodness-of-fit tests, calibration curves, decision curves, etc.; three studies (15, 23, 27) conducted internal validation, with the Bootstrap method as the main method, two studies (16, 20) carried out external validation, and two studies (32, 33) used a combination of internal and external validation for model evaluation. The model performance and presentation form were shown in **Table 3**.

**Table 3:** Model performance and presentation form.

Studies	Performance of the model		Model validation methods	Model presentation form
	Degree of discrimination	Degree of calibration		
Mao CC (12)	D: C-index = 0.736	Not mentioned	Not mentioned	Nomogram
Chen L (13)	D: AUC = 0.987 (95%CI: 0.948–0.999)	H-L test	Not mentioned	Risk scoring system
Ye GQ (14)	D: AUC = 0.702	Not mentioned	Not mentioned	Risk scoring system
Hu LS (15)	D: AUC = 0.945 (95%CI: 0.871–0.971)	H-L test, calibration curve, decision curve analysis	Bootstrap method was used for internal validation	Nomogram
Cagigas (16)	D: (LR) AUC = 0.812 (95%CI: 0.746–0.877) D: (BN) AUC = 0.837 (0.769–0.904)	Not mentioned	Single-center external validation	Bayesian network diagram
Sun C (17)	D: AUC = 0.780 (95%CI: 0.710–0.840)	H-L test	Not mentioned	Risk scoring system
Liu SF (18)	D: AUC = 0.813 (95%CI: 0.713–0.913)	H-L test	Not mentioned	Risk score formula
Pei G (19)	D: AUC = 0.931 (95%CI: 0.815–0.971)	Calibration curve, decision curve analysis	Not mentioned	Nomogram
Zhang Y (20)	D: AUC = 0.745 (95%CI: 0.650–0.840) V: AUC = 0.736 (95%CI: 0.602–0.871)	Not mentioned	Single-center external validation	Risk score formula + Nomogram
Liu J (21)	D: AUC = 0.851 (95%CI: 0.772–0.930)	Not mentioned	Not mentioned	No specific model was given
Luo J (22)	Not mentioned	Not mentioned	Not mentioned	Bayesian network diagram
Wu TL (23)	D: AUC = 0.905 (95%CI: 0.855–0.956)	Calibration curve, decision curve analysis	Bootstrap method was used for internal validation	Nomogram
Yang L (24)	D: AUC = 0.857 (95%CI: 0.744–0.987)	Not mentioned	Not mentioned	No specific model was given
Zhang WB (25)	D: AUC = 0.846 (95%CI: 0.809–0.878)	H-L test	Not mentioned	Risk score formula
Chen YG (26)	D: AUC = 0.872 (95%CI: 0.817–0.916)	H-L test	Not mentioned	Risk score formula
Jin J (27)	D: AUC = 0.771 (95%CI: 0.699–0.842)	Calibration curve, decision curve analysis	Bootstrap method was used for internal validation	Nomogram
Luo C (28)	D: AUC = 0.749 (95%CI: 0.676–0.822)	H-L test, decision curve analysis	Not mentioned	Nomogram
Shi ZQ (29)	D: AUC = 0.828 (95%CI: 0.764–0.880)	H-L test, calibration curve analysis	Not mentioned	Risk score formula + Nomogram
Sun Y (30)	D: AUC = 0.883 (95%CI: 0.851–0.911)	H-L test	Not mentioned	Risk score formula
Wang S (31)	D: AUC = 0.880 (95%CI: 0.779–0.981)	H-L test	Not mentioned	Nomogram
Liu CQ (32)	D: AUC = 0.968 (95%CI: 0.948–0.988) V: AUC = 0.926 (95%CI: 0.906–0.980)	Calibration curve, decision curve analysis	Bootstrap method was used for internal validation + Single-center external validation	Nomogram
Yu X (33)	D: AUC = 0.933 (95%CI: 0.874–0.895) V: AUC = 0.951 (95%CI: 0.843–1.000)	Calibration curve, decision curve analysis	Bootstrap method was used for internal validation + Single-center external validation	Nomogram

D, Development; V, Validation; LR: logistic regression; BN, Bayesian network; CI, Confidence Interval; AUC, the rea under the receiver operating characteristic curve; C-index, Index of concordance; H-L test, Hosmer-Lemeshow goodness-of-fit test.

### 3.5 Evaluation of risk of bias and applicability

The 22 included studies (12–33) all showed a high risk of bias. Study population domain: 19 studies (13, 15, 16, 18–33) were at high risk of bias because their data came from retrospective studies, there was a bias for sample size inclusion, and there may be recall bias. Predictor domain: 6 predictors (18, 22, 26, 28, 30, 31) were rated as “high risk” because the source of definition of the predictors was not clearly stated, which may have resulted in biased model performance, while all other studies were at low risk of bias. Outcome domain: 4 studies were rated as high risk of bias: 2 studies (13, 31) were rated as “high risk of bias” because of partial duplication of predictors and outcome indicators, and 2 studies (21, 32) did not clearly state the definition of the outcome, which might lead to biased model performance; Analytic domain: all the studies were rated as high risk of bias, 20 studies (12–15, 17–21, 23–33) had insufficient outcome events, with the number of events per variable (EPV) <20; 14 studies (12, 14, 15, 18, 19, 22–24, 26–31) were partial or full discretization of continuous variables; 2 studies (13, 21) screened predictor variables based on univariate analysis only; 7 studies (12, 14, 17, 19, 20, 27, 28) did not mention the treatment of missing data; 7 studies (12, 14, 16, 20–22, 24) were missing the model calibration test, and 15 studies (12–14, 17–19, 21, 22, 24–26, 28–31) were not validated. In the applicability evaluation, three studies (18, 23, 33) in the study population domain were rated as high applicability risk for restricting their study to a specific population. Five studies (14–16, 19, 31) in the outcome domain were judged to be unclear because the source of the definition of the outcome indicator was not reported. The remaining studies had good applicability.

**Figure 2:**
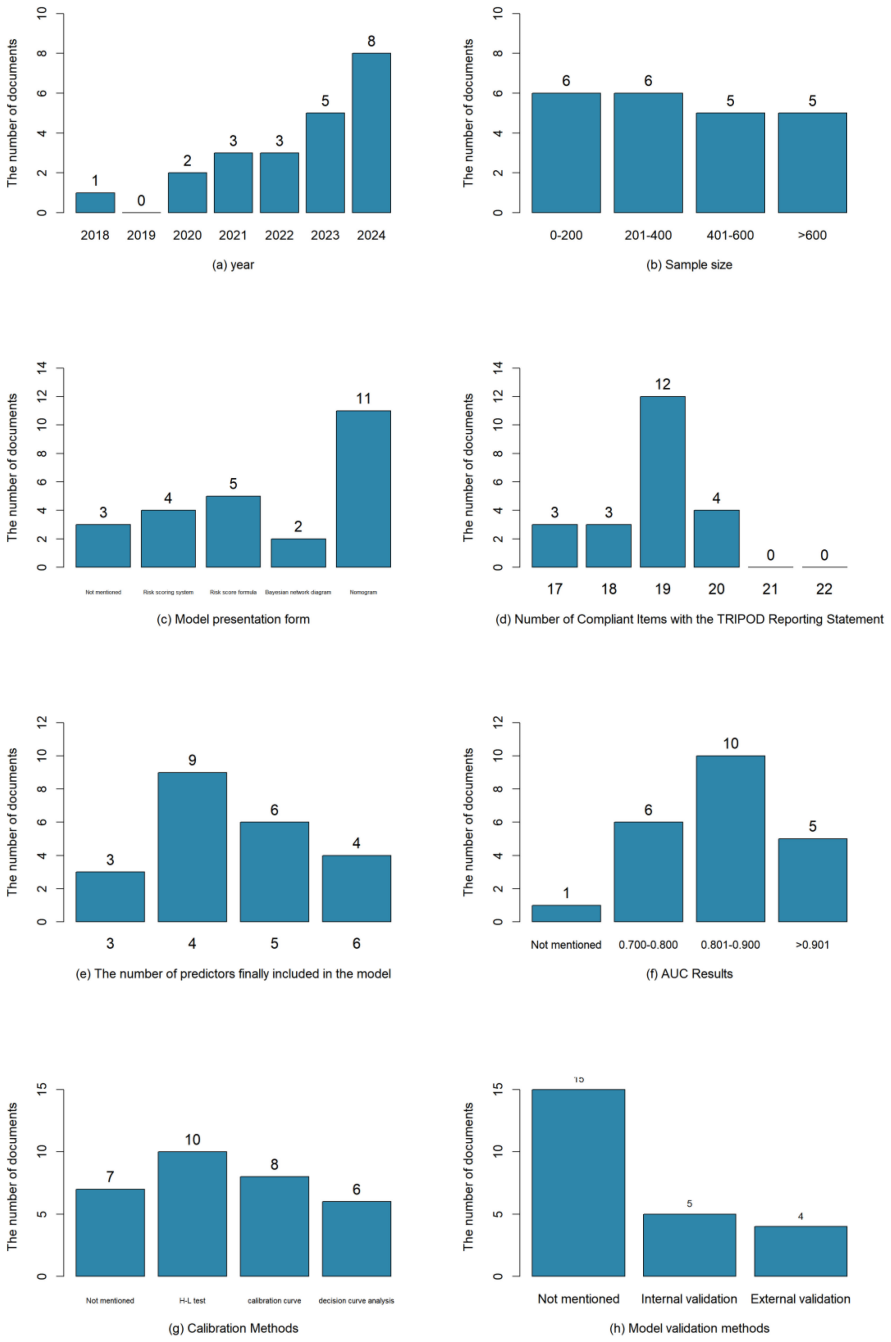
Visual presentation of the basic information included in the study.

Studies with higher reporting quality may reflect more rigorous validation methods and more reliable performance estimates. In this study, an assessment of the 22 included studies (12–33) was conducted against the TRIPOD reporting guidelines. Results showed that the reporting quality of the studies varied, with TRIPOD compliance ranging from 77.3% (17/22) to 90.9% (20/22). The commonly underreported items included "explaining how the study sample size was determined" (Item 8) and "providing supplementary materials and information, such as study protocols, web-based calculators, and datasets" (Item 21). The results of the literature evaluation were shown in **Table 4**. The visual presentation of the basic information of the included studies is shown in **Figure 2**.

**Table 4:** Quality evaluation of literature.

Included studies (first author/year of publication)	Risk of bias assessment				Assessment of applicability			Overall evaluation		Assessment of the TRIPOD reporting guideline	
	Subjects	Predictors	Results	Analysis	Subjects	Predictors	Results	Risk of bias	Applicability	Compliance (n/22, %)	Missing items
Mao CC (12)	L	L	L	H	L	L	L	H	L	19/22 (86.4%)	8, 9, 16
Chen L (13)	H	L	H	H	L	L	L	H	L	20/22 (90.9%)	8, 21
Ye GQ (14)	L	L	L	H	L	L	N	H	N	17/22 (77.3%)	5, 8, 9, 16, 21
Hu LS (15)	H	L	L	H	L	L	N	H	N	19/22 (86.4%)	8, 18, 21
Cagigas (16)	H	L	L	H	L	L	N	H	N	17/22 (77.3%)	8, 13, 18, 21, 22
Sun C (17)	L	L	L	H	L	L	L	H	L	19/22 (86.4%)	8, 9, 13
Liu SF (18)	H	H	L	H	H	L	L	H	H	19/22 (86.4%)	6, 8, 21
Pei G (19)	H	L	L	H	L	L	N	H	N	20/22 (90.9%)	8, 9
Zhang Y (20)	H	L	L	H	L	L	L	H	L	19/22 (86.4%)	8, 9, 22
Liu J (21)	H	L	H	H	L	L	L	H	L	19/22 (86.4%)	8, 18, 21
Luo J (22)	H	H	L	H	L	L	L	H	L	17/22 (77.3%)	8, 16, 18, 21, 22
Wu TL (23)	H	L	L	H	H	L	L	H	H	19/22 (86.4%)	8, 21, 22
Yang L (24)	H	L	L	H	L	L	L	H	L	18/22 (81.8%)	8, 14, 15, 21
Zhang WB (25)	H	L	L	H	L	L	L	H	L	19/22 (86.4%)	8, 18, 21
Chen YG (26)	H	H	L	H	L	L	L	H	L	18/22 (81.8%)	1, 8, 21, 22
Jin J (27)	H	L	L	H	L	L	L	H	L	19/22 (86.4%)	8, 9, 21
Luo C (28)	H	H	L	H	L	L	L	H	L	18/22 (81.8%)	8, 9, 18, 21
Shi ZQ (29)	H	L	L	H	L	L	L	H	L	19/22 (86.4%)	8, 18, 21
Sun Y (30)	H	H	L	H	L	L	L	H	L	19/22 (86.4%)	8, 14, 21
Wang S (31)	H	H	H	H	L	L	N	H	N	20/22 (90.9%)	8, 21
Liu CQ (32)	H	L	H	H	L	L	L	H	L	20/22 (90.9%)	1, 8
Yu X (33)	H	L	L	H	H	L	L	H	H	19/22 (86.4%)	8, 21, 22

H, High risk; L, Low risk; N, Not clear.

### 3.6 Meta-analysis results

**Figure 3:**
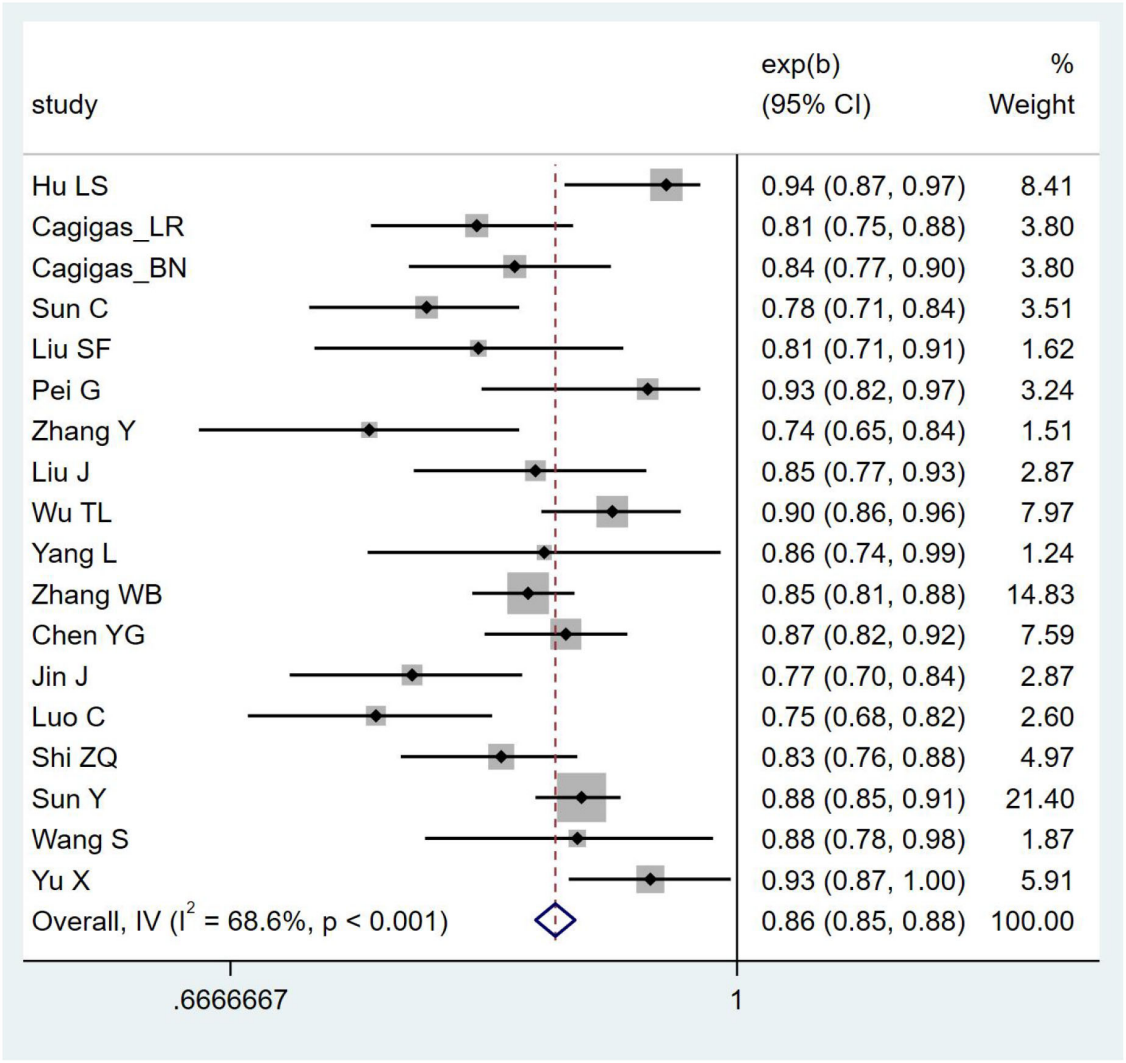
Forest plot of the random effects meta-analysis of combined AUC estimates.

Due to insufficient details reported by the models of some included studies, only 19 studies were ultimately eligible and included in the meta-analysis. The postoperative IAI prediction models for patients with gastrointestinal malignant tumors exhibited high heterogeneity, so a random-effects model was used for analysis [*I*²=88.4%, *P <*0.001, AUC = 0.917 (0.906–0.927)]. After applying the one-by-one exclusion method, the results were [*I*²=68.6%, *P <*0.001, AUC = 0.865 (0.851–0.878)]. The forest plot of AUCs is shown in **Figure 3**.

## 4 Discussion

Patients with malignant tumors of the digestive system have a high incidence rate of postoperative IAI, and this postoperative IAI is closely associated with prolonged hospital stay, decreased quality of life, and increased patient mortality (19). Risk prediction models for postoperative IAI can identify high-risk groups at an early stage and promptly provide preventive and interventional measures to reduce its incidence and decrease adverse outcomes. Furthermore, this study systematically reviewed current research on IAI risk prediction models for patients with digestive system tumors. The prediction models included in the 22 selected studies exhibited good predictive performance and could accurately identify high-risk patients for postoperative IAI among those with malignant digestive system tumors. However, their overall methodological quality and reporting standardization are concerning. Assessment using the Prediction Model Risk of Bias Assessment Tool (PROBAST) revealed that all included studies had a high risk of bias, with particular deficiencies in model validation, calibration assessment, and missing data handling.

During the model development phase, most of the studies included in this review were of retrospective design. This design cannot guarantee the accuracy of data collection and is prone to interference from existing results, which in turn increases model heterogeneity. In the future, cohort studies or nested case-control studies should be conducted to reduce the impact of information bias on model construction. Regarding variable selection, the traditional approach of univariate followed by multivariate analysis is commonly adopted. This method fails to comprehensively evaluate the interactions and inherent relationships among candidate variables, and it also tends to overlook important variables. It is recommended that new methods be adopted for variable selection in future studies, in conjunction with clinical practice. Examples of such methods include LASSO regression, Ridge regression, and ElasticNet regression (34). It is significantly superior to traditional variable selection methods in handling multicollinearity, controlling overfitting, and objectively selecting variables. It helps improve the accuracy of selection and contributes to building a more concise, stable, and clinically practical postoperative IAI prediction model. Furthermore, poor handling of continuous variables and insufficient outcome events corresponding to predictors (EPV <20) have further increased the risk of model overfitting and undermined the stability and validity of the models. In future research, every effort should be made to maintain data continuity during the model construction process, and emphasis should be placed on conducting large-sample exploratory studies. By providing a more extensive data foundation, researchers will be able to capture more variability and potential confounding factors.

During the model evaluation and reporting phase, some studies only evaluated discriminative ability and did not report calibration, resulting in incomplete performance assessment of clinical prediction models. Scholars worldwide should conduct timely calibration and report relevant results after model development; this not only facilitates the comparison of established risk prediction models but also supports their clinical translation. On the other hand, most models have not undergone effectiveness validation, and their generalization ability remains unknown. The internal and external validation of models occupies a core position in the transition from theoretical model construction to practical clinical application, and is crucial to the overall stability and applicability of the models (35). Therefore, in subsequent research, it is necessary to strengthen validation efforts and exercise strict control over this process. Finally, unclear reporting or improper handling of missing data can also seriously undermine the transparency and reproducibility of the study. It is recommended that missing data be reported and addressed using weighting methods or imputation methods; this can effectively reduce the negative impact of missing values on statistical analysis and model reliability, and achieve minimum bias (36). The aforementioned flaws collectively form the basis for determining "high risk of bias" in the PROBAST assessment.

While this study, through a meta-analysis, found that the pooled AUC of the included studies was 0.865 (95% CI: 0.851–0.878), indicating good discriminative ability of the models, AUC only reflects the discriminative power of a model and does not indicate the accuracy of predicted probabilities. If a model does not report or improve calibration, it may mislead clinical practice in clinical decision-making (e.g., threshold selection, risk stratification). In addition, most studies lack external validation, making it impossible to confirm the generalizability of the models. Based on the above reasons, we adopt a conservative interpretation of the relatively high AUC values reported in the original studies. We suggest that these models should undergo independent validation in external and more representative cohorts, and report calibration curves and decision curve analyses, before their clinical applicability can be further evaluated.

The methodological flaws revealed in this study are not an isolated phenomenon in the development of prediction models within the oncology field. A systematic review that applied the PROBAST framework to treatment-related toxicity models in cancer patients found that most models carried a high risk of bias, with external validation being severely lacking (37). Similarly, in a review of colorectal cancer disease risk prediction models, researchers also emphasized the problem that calibration is commonly overlooked (38). Future researchers should continue to explore the risk factors for postoperative IAI in patients with digestive system tumors, while focusing on the following key points: optimizing research design and conducting prospective multicenter studies; scientifically handling missing data, with detailed descriptions of the proportion of missing data and the methods used for processing; performing internal and external validation to evaluate the extrapolation performance of the model; and strictly adhering to the methodological details of the TRIPOD Statement and PROBAST tool to improve the standardization of reporting.

Compared with these studies, the unique added value of this study lies in that it for the first time focuses on postoperative IAI in patients with digestive system tumors—a specific and high-risk complication—and summarizes the high-frequency predictors across various models. The length of surgery, ALB, comorbid diabetes mellitus, length of drain retention, and age were the most frequently occurring predictors, and future modeling could focus on the above five factors: A significant increase in the length of surgery showed a strong association with a rising risk of postoperative IAI (24–26, 33). The reason for this is that prolonged exposure of the surgical area provides an opportunity for bacterial colonization. At the same time, the prolonged pulling and compression of the retractor on the tissues of the body results in poor blood circulation, which in turn weakens the body's antimicrobial capacity and significantly increases the probability of infection (39). Therefore, during the surgical operation, it was important to uphold the principle of prudence and meticulousness, under the premise of ensuring surgical safety as far as possible, shorten the duration of the operation, so as to effectively reduce the probability of postoperative IAI; The Chinese Guidelines for the Diagnosis and Treatment of Abdominal Infections pointed out that (40), the low level of ALB was a risk factor for the death of patients with IAI, and as a core indicator reflecting the nutritional status of the patient, it was important for patients with gastrointestinal tumors to have the best possible nutritional status before and after the operation. Tumor patients' preoperative and postoperative nutritional intervention plan development has a good guiding value. It was recommended to routinely screen patients' ALB levels, and clinical personnel can instruct patients to eat high-calorie and high-protein foods according to their daily dietary habits to ensure that they have sufficient and comprehensive nutritional needs, and if necessary, provide patients with nutritional support measures, such as intravenous albumin infusion, if the index was at a lower level or continues to decline; eight studies (15, 18, 24–29) have identified combined diabetes as a predictive factor. Diabetes mellitus was listed as a predictor, because diabetes mellitus patients were in negative nitrogen balance for a long time, the rate of catabolism was greater than anabolism, which affected the synthesis of related immune factors and makes the body immune function disorders. In addition, diabetes can affect the neutrophil chemotaxis and phagocytosis of the patient's body, which made the body's immune system reduce the clearance of pathogenic bacteria. At the same time, the persistent high glucose state was conducive to the colonization and attachment of pathogenic bacteria, which in turn leads to a further increase in the risk of abdominal cavity infection (41, 42). For patients with impaired glucose tolerance and impaired fasting glucose regulation, it was necessary to strengthen blood glucose monitoring, scientifically formulate the blood glucose management process and intervention strategies, so as to effectively control blood glucose levels and reduce the occurrence of postoperative IAI; in digestive system tumor resection, due to the large surgical trauma, a large amount of exudate was generated in the postoperative period, and the main purpose of the abdominal cavity drainage tube is to drain exudate in the postoperative period. Four studies (24, 26, 28, 29) took the duration of drainage tube indwelling as a predictor to analyze. The reasons for this are analyzed as follows, if left in place for too long, secretions and contents may overflow from the side of the tube, inducing infection around the drainage tube and retrograde infection of the abdominal cavity, increasing the possibility of postoperative IAI in patients (43). Despite the aforementioned possibility, all four studies were retrospective in design. It is difficult to accurately define its time sequence and causal logic. In addition, some of these studies failed to clearly document the start time of the indwelling duration, which may introduce the bias of "passive prolongation of indwelling following the onset of IAI". For example, if infection leads to increased exudation, clinicians may delay extubation; in such cases, the indwelling duration is actually a consequence of IAI rather than its cause. Therefore, the current evidence only demonstrates a strong correlation between the two factors, and the causal relationship requires further verification through prospective cohort studies. In clinical practice, individualized extubation strategies can be formulated based on dynamic assessments (e.g., daily monitoring of the characteristics of drainage fluid), rather than solely relying on thresholds for indwelling duration. Clinicians should prejudge the amount and nature of postoperative exudate in advance to determine whether an abdominal drain must be left in place and to choose the appropriate type and size of drain, so that the drain can be correctly placed during surgery to ensure that it is in the optimal drainage position. After surgery, the timing of tube removal should be scientifically evaluated; age was closely related to the function of the patient's organs, tolerance and immune function, and elderly patients have declining organ function, reduced tolerance and low immune function. In addition, surgery causes more significant stressed in elderly patients, which further inhibits their immune function and substantially increases the risk of postoperative IAI (44). In future clinical work, attention should be paid to this group of people, and targeted immunity enhancement should be carried out as early as possible, and early screening of IAI-related clinical indicators in high-risk groups should be carried out, so as to reduce the incidence of postoperative IAI.

Regarding the direct clinical application of the models, we must adopt a cautious attitude. Among the 22 models included in this study, we found that only 11 provided user-friendly nomograms, while most models remain at the stage of formulas in papers—this greatly limits the clinical translation of prediction models. Therefore, scholars in various countries can transform the model into technological forms, such as web calculators and APPs, and grade the risk level, so that clinicians can implement targeted and personalized stratified prevention and management strategies. When researchers apply the prediction model to clinical work, they should pay attention to combining the individual characteristics of high-risk groups, and optimize and continuously calibrate the prediction model in a timely manner, which will not only help healthcare workers to implement appropriate interventions for high-risk groups to ensure the treatment outcome of the patients, but also alleviate the economic burden of the patients, and reduce the overall cost of healthcare.

To summarize, though research on risk prediction models for postoperative IAI in patients with digestive system tumors has begun, it is far from mature. Future research urgently needs to make breakthroughs in the following aspects: In the literature included in this study, logistic regression was mostly used for modeling, only 2 studies (16, 22) of the included studies incorporated Bayesian networks. The differences in the application of these two approaches reflect both the practical utility and methodological challenges of clinical prediction models. As a type of generalized linear models, logistic regression has the advantages of strong interpretability and fast training speed, but it was difficult to fit the real distribution of multidimensional complex data, and it may have an impact on the predictive performance of the model when there are data deficiencies or multicollinearity (45, 46). The Bayesian network method, based on Bayes' theorem, is more flexible in handling nonlinear relationships and missing data. However, it requires substantial data support, has poor interpretability, and the model visualization results pose a high barrier to understanding for non-statistical professionals—all of which limit its clinical popularization. Currently, the popularity of developing clinical prediction models using machine learning approaches is rapidly increasing, machine learning techniques can automatically analyze a large amount of data and deeply mine the correlation and logic between the data, so the models created can fit the data more closely to the real situation. It was recommended that future researchers should select appropriate machine learning algorithms based on the pathophysiological mechanisms of IAI after surgery in patients with digestive malignancies by digging deeper and clarifying the potential risk factors, and construct multiple models, conduct comparisons among them, and select the optimal model to achieve better prediction performance. Construct multiple models, conduct comparisons among them, and select the optimal model to achieve better prediction performance. At this stage, several studies have obtained data sources from structured data of electronic medical record systems. From the perspective of data-driven research, complete and high-quality medical datasets have a central position in the training process of risk prediction models (47), and in tuture, the clinical healthcare information system structured on the core of the electronic medical record can be improved and optimized in depth, so as to build a solid data foundation for the construction of high-precision risk prediction models, thus realizing the accurate prediction and assessment of disease risk. In addition, the number of externally validated studies in the literature included in this study is relatively small, and their representativeness and extrapolation need to be verified. In the future, researchers in various countries should conduct multi-center and large-sample application validation studies globally to improve the generalization ability and applicability of the model, and effectively promote the application of the model on the ground.

This study also has certain limitations: First, the study results are only based on currently available model studies and have inherent limitations in terms of regional differences, population applicability, and other aspects. Second, the included literature is limited to Chinese and English, and high-quality model studies in other languages may have been omitted. Finally, due to significant heterogeneity in study subjects and exclusion criteria, it was not possible to conduct a subgroup analysis or sensitivity analysis, and only qualitative descriptions were performed.

## 5 Conclusion

In summary, a total of 22 cases were included in this systematic review, and 23 prediction models were constructed. The results showed that the postoperative IAI prediction models for patients with digestive system tumors demonstrated good performance and applicability, but there was a high risk of bias and heterogeneity. Given this, it is recommended that scholars worldwide focus on large-sample, prospective, and multicenter external validation studies, strictly adhere to the TRIPOD Statement, and standardize study design and reporting processes. Furthermore, future studies may also select appropriate machine learning algorithms, conduct internal and external validation, and further evaluate the efficacy and feasibility of the models in clinical practice, thereby providing more reliable support for clinical decision-making.
